# Kaposi's sarcoma in a patient with pemphigus vulgaris mimicking exacerbation of pemphigus

**DOI:** 10.1002/cnr2.1815

**Published:** 2023-03-31

**Authors:** Kamran Balighi, Maryam Ghiasi, Zeinab Aryanian, Zahra Nikyar, Parvaneh Hatami

**Affiliations:** ^1^ Autoimmune Bullous Diseases Research Center Tehran University of Medical Sciences 1199663911 Tehran Iran; ^2^ Department of Dermatology, School of Medicine, Razi Hospital Tehran University of Medical Sciences 1199663911 Tehran Iran; ^3^ Department of Dermatology Babol University of Medical Sciences 47176_47745 Babol Iran; ^4^ Department of Dermatology, School of Medicine, Pediatric Dermatology Fellowship, Razi Hospital Tehran University of Medical Sciences 1199663911 Tehran Iran

**Keywords:** iatrogenic, immunosuppressive agents, Kaposi's sarcoma, pemphigus vulgaris, unusual presentation

## Abstract

**Background:**

Kaposi's sarcoma (KS) is a rare multifocal angiogenic tumor often seen in immunocompromised setting such as acquired immunodeficiency syndrome (AIDS) or organ transplantation recipients. Pemphigus vulgaris (PV) is a rare blistering disorder with mucocutaneous involvement for which immunosuppressive therapy has long been the core of treatment. Iatrogenic form of KS has been reported infrequently in pemphigus patients as a result of long‐term immunosuppressive therapy.

**Case:**

We describe a 39‐year‐old male patient with confirmed diagnosis of PV who developed KS after receiving immunosuppressive agents for his pemphigus. KS was initially localized to the oral cavity with features mimicking exacerbation of his pemphigus.

**Conclusion:**

This interesting case of KS suggests that dermatologists visiting patients with pemphigus with discomfort in the oral cavity should have a high degree of awareness and consider other differential diagnoses along with merely an exacerbation of PV.

## 1 INTRODUCTION

Pemphigus vulgaris (PV) is a rare blistering disorder with mucocutaneous involvement characterized by producing autoantibodies against desmogleins (Dsg) which play an important role in maintenance of cell‐to‐cell adhesion.^1^ Immunosuppressive therapy has long been the core of treatment for pemphigus,^2^, ^3^, ^4^, ^5^ though might lead to some serious adverse effects such as susceptibility to neoplastic events.^6^, ^7^

Kaposi's sarcoma (KS) is a rare multifocal angiogenic tumor usually presents with violaceous patches and papules of the skin or mucosal area and often seen in immunocompromised setting such as acquired immunodeficiency syndrome (AIDS) or organ transplantation recipients.^8^

Iatrogenic form of KS has been reported infrequently in patients with pemphigus due to long‐term immunosuppressive therapy.^9^

Here, we present a case of KS, presented in an Iranian known case of PV with features might have led to misdiagnosis of exacerbation of his pemphigus.

## 2 CASE REPORT

A 39‐year‐old male patient, had been diagnosed with mucocutaneous PV 15 months earlier, visited our outpatient pemphigus clinic at Razi Hospital affiliated to the Tehran University of Medical Sciences, Tehran, Iran for regular follow‐up visit on February 2021, complaining of a persistent sore throat despite considerable improvement of his cutaneous lesions.

He was firstly presented with painful oral ulcers and widespread cutaneous erosions mainly over truncal area for 3 months. Histopathologic and DIF examinations stablished the diagnosis of PV. His disease had initially been controlled with 60 mg/day prednisolone which tapered to 30 mg/day during 1 month and rituximab (one cycle consisting of one infusion of 500 mg every week for 4 weeks) was added to his therapeutic regimen leading to a considerable clinical improvement. Hence, prednisolone was rapidly tapered to 10 mg/day in 3 months because of his cushingoid appearance and also achieving partial clinical remission. The Pemphigus Disease Area Index (PDAI) score of cutaneous and mucosal lesions from 11 and 10 at the baseline, declined to 0 and 3 at the fifth month of treatment, respectively.

The patient had also taken Isoniazide (INH) and vitamin B6 as tuberculosis prophylaxis due to positive skin tuberculin test (induration: 13 mm). Patient was otherwise healthy.

By the time, the prednisolone dosage was lowered to 7.5 mg/day and cutaneous PDAI score was 0, but he was still complaining of a persistent odynophagia and dysphonia.

**FIGURE 1:**
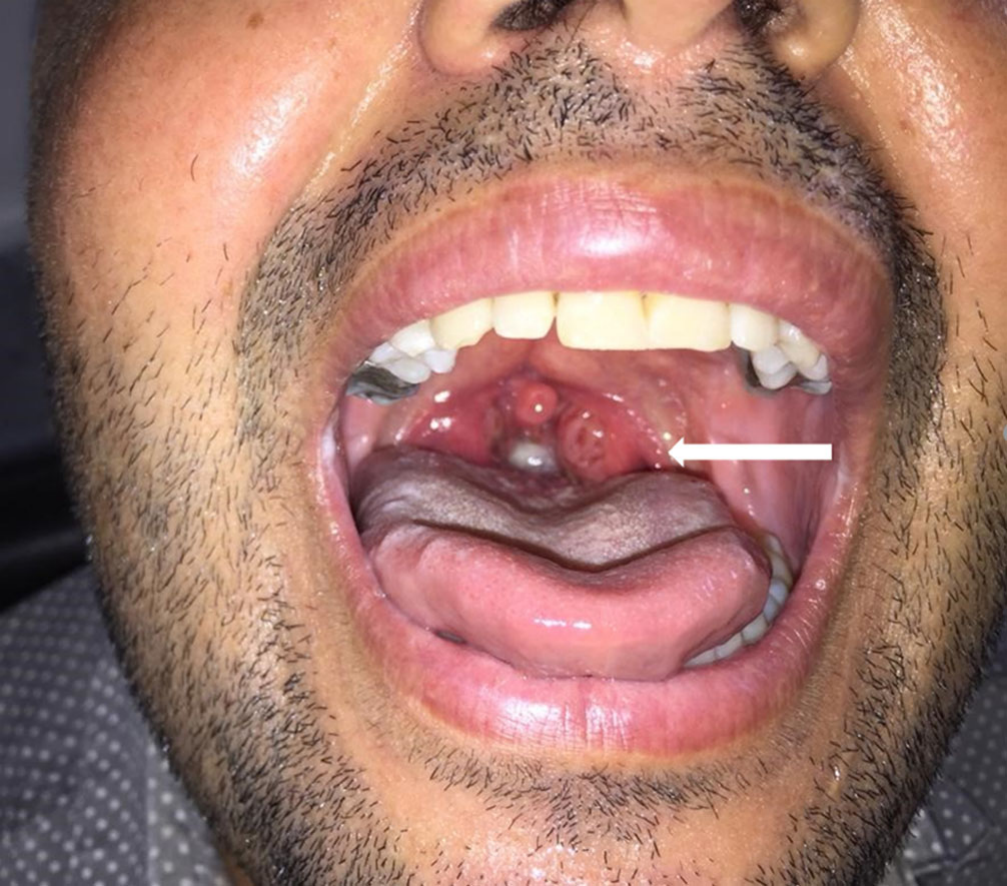
The erythematous—violaceous nodule with erosive surface at the end of lingual area.

On physical examination, the patient had generally good health and no remarkable finding was noted except for mild sore throat and an erythematous nodule with erosive surface at the end of lingual area (Figure 1). Although it initially was assumed to be a vegetative lesion of his pemphigus, but the unusual morphological appearance of lesion including its mild violaceous hue, led us to consider the possibility of a secondary pathologic process in this area.

MRI of cervical soft tissue revealed an isosignal 23 × 73 mm mass at oropharyngeal region on epiglottis which was enhanced after contrast injection. Pressure effect on airway was also noted.

**FIGURE 2:**
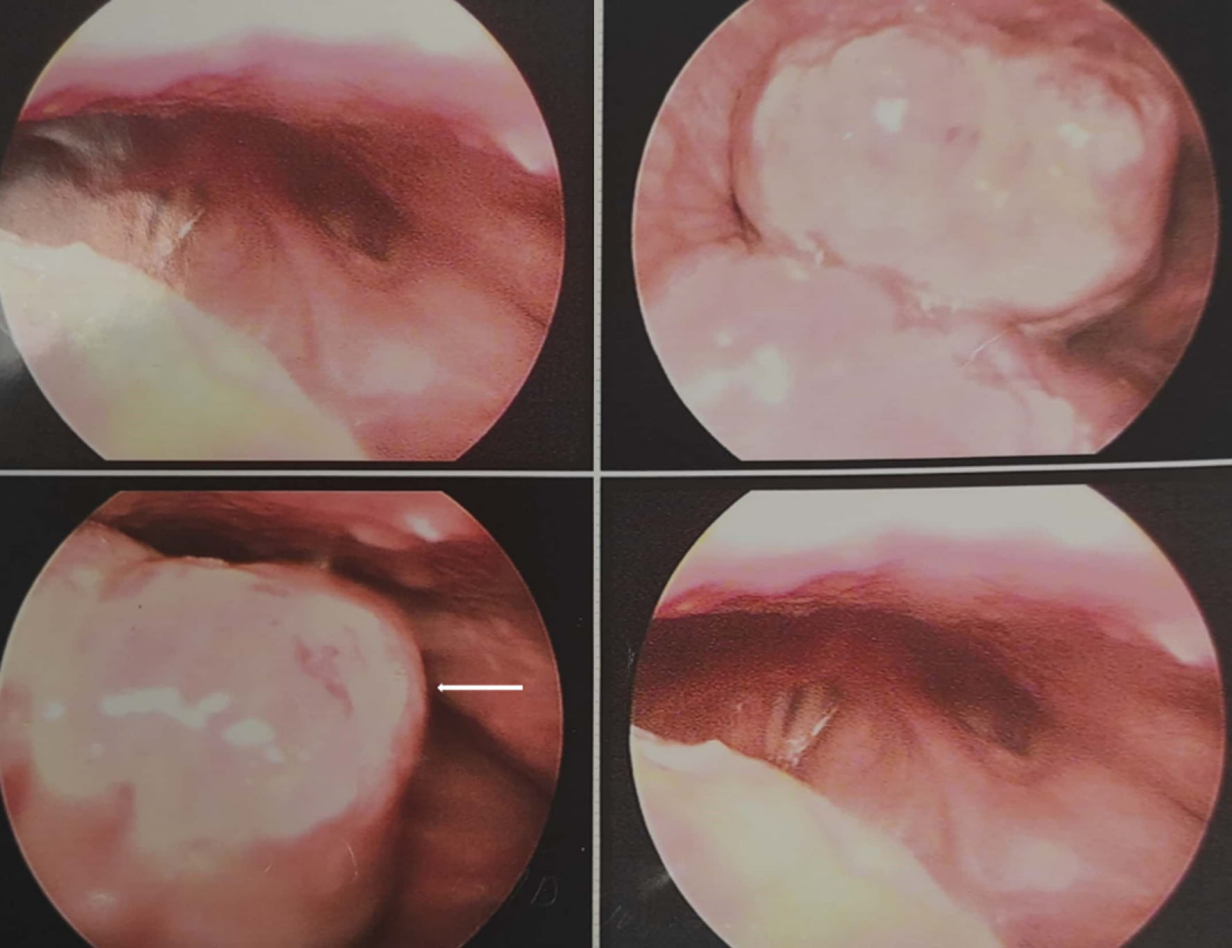
A view of vocal cords during laryngoscopy. A large mass was notified at the base of tongue.

**FIGURE 3:**
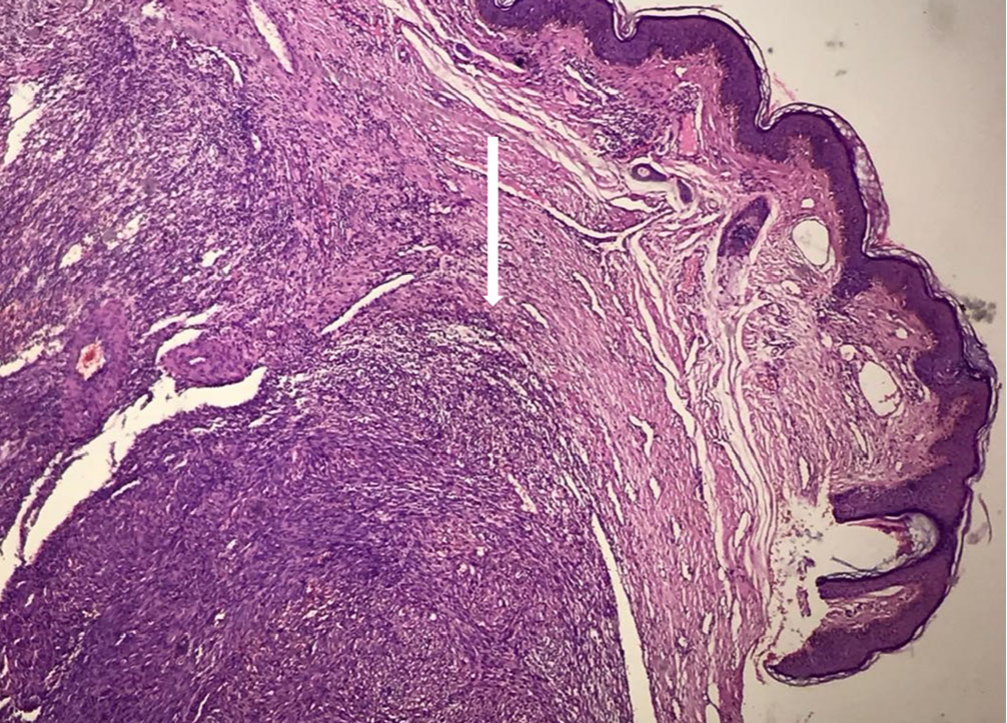
A nodular spindle cell neoplasm with hyper vascularity consisted of slit‐like vascular spaces formed by spindled endothelial cells with minimal to moderate atypia (H&E× 40).

**FIGURE 4:**
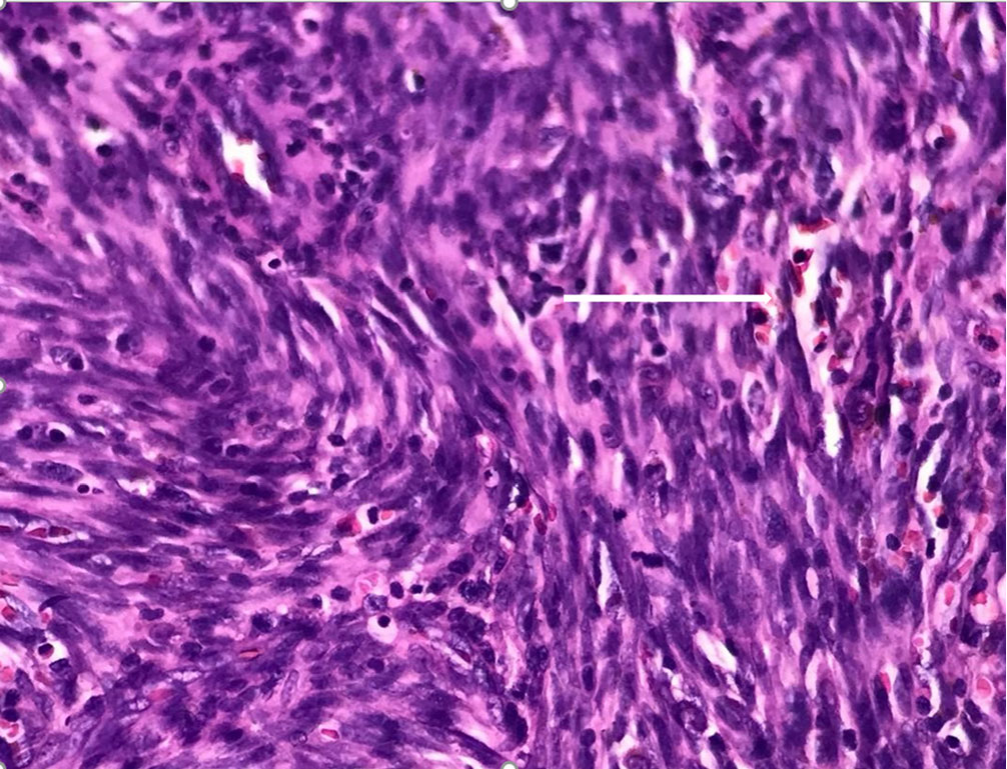
Nodular and vascular/ spindle cell proliferation with slit‐like spaces filled with erythrocytes, hyaline globules and peripheral dilated vessels consistent with the diagnosis of KS (H&E× 100).

**FIGURE 5:**
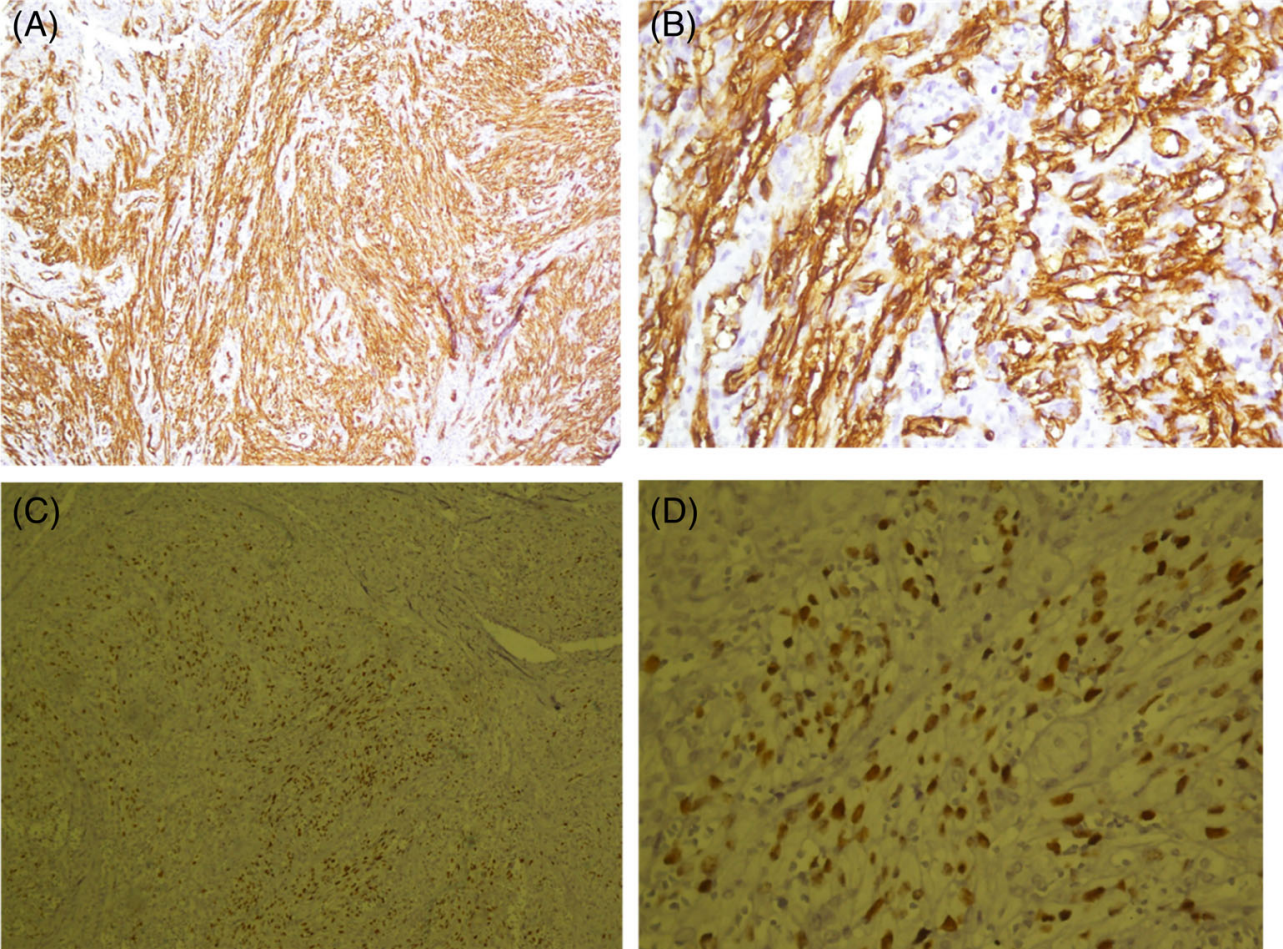
Immunohistochemistry examination of tissue sample for CD34 (A and B) and HHV‐8 (C and D) 6.

The patient was referred to the Otolaryngology Department, where the nodule was totally excised after a laryngoscopic evaluation in which a large mass was detected at the base of tongue (Figure 2). Histopathologic examination of the tissue showed nodular and vascular/ spindle cell proliferation with slit‐like spaces filled with erythrocytes, hyaline globules and peripheral dilated vessels which were consistent with the diagnosis of KS (Figures 3 and 4). IHC study for CD34 and HHV‐8 (Human Herpes Virus 8) confirmed the diagnosis (Figure 5). DNA sequence analysis for HHV‐8 on tissue samples or evaluating of HHV‐8 antibody on the patient's serum was not performed, but serology for HIV‐1 was negative.

During systemic evaluation, no history of opportunistic infections, B symptoms or any sign or symptom regarding visceral involvement of KS was noted.

Taking into consideration the clinical complete remission of PV in patient as well as the low values of anti‐Dsg1, 3 (1 and 1.6 IU/mL, respectively), we decided to taper off the prednisolone.

**FIGURE 6:**
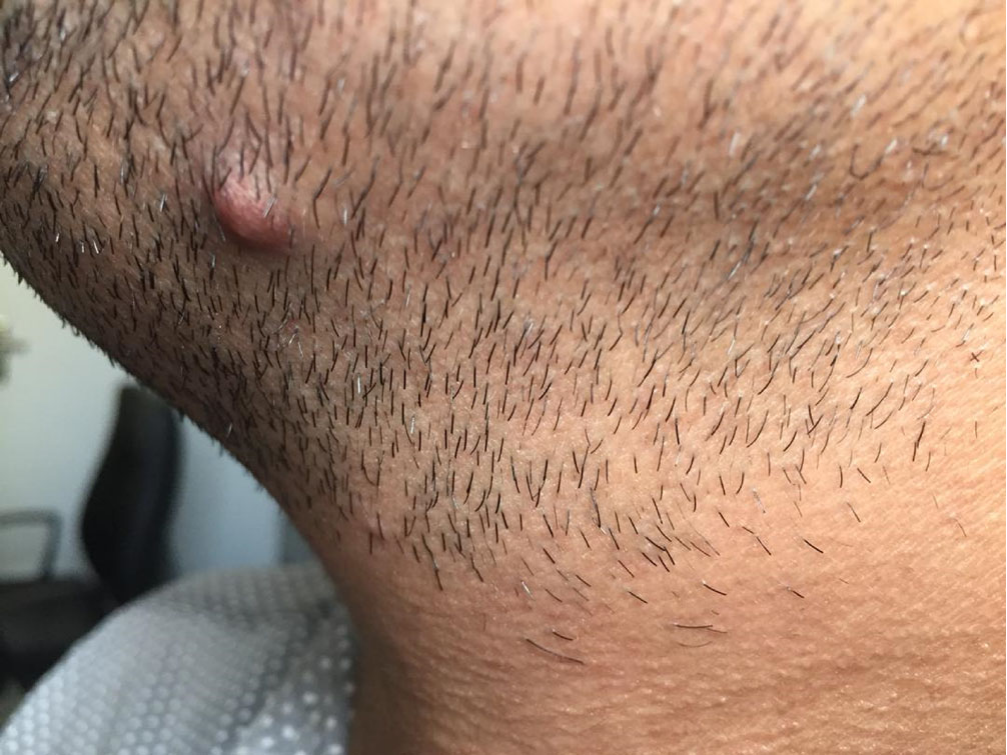
Second lesion of KS appeared 1 month later on the patient's cheek.

One month later, two other nodular violaceous lesions on his cheek and ankle were appeared (Figure 6) which were totally excised and their histopathologic findings were again consistent with KS including the expansion of dermis by a solid tumor nodule consisted of fascicles of relatively monomorphic spindled cells, with slit‐like vascular channels containing erythrocytes. After consultation with oncology department, a watchful follow‐up of patient instead of initiation of systemic chemotherapy was recommended due to the fact that all of three lesions were being previously excised and patient was otherwise healthy.

Any occurrence of KS lesion has not been observed until now (21 months after excision of the last lesion) and the patient did not describe any relapses of PV or KS lesions.

## 3 DISCUSSION

KS is an angioproliferative neoplasm of low‐grade malignant potential.^10^ Its exact etiopathogenesis is not completely understood, but impaired immunity and HHV‐8 virus infection along with angiogenic milieu due to inflammatory cytokines could lead to the disease development.^11^, ^12^

It is hypothesized that altered immunity weakens the immunological surveillance and leads to reactivation of a latent HHV‐8 infection in genetically susceptible patients.^8^

KS is classified into four types: classic, endemic, immunosuppression‐associated or iatrogenic and AIDS‐associated or epidemic.^13^

Association of pemphigus (mainly PV) and KS has been previously reported.^14^ In fact, pemphigus‐associated KS is regarded as an iatrogenic disorder, develops after receiving immunosuppressive therapy, mainly corticosteroids.^14^, ^15^, ^16^ However, rituximab‐induced KS in HIV‐negative patients (but not pemphigus patients), though as an extremely rare condition, have been previously reported.^17^, ^18^ As mentioned before, rituximab had been administered for our patient in the course of his pemphigus treatment which might be considered among potential triggers for the development of his KS lesions.

Iatrogenic KS often has similar clinical features to the AIDS‐associated KS including fulminant course with widespread lesions, tendency to visceral involvement^19^ and poor prognosis.^20^, ^21^ Tourlaki et al.^22^ recently reported that 30% of reported cases of pemphigus‐associated KS had visceral involvement and 35% died briefly after diagnosis. A relatively fair course of KS lesions was noted in our case after surgical excision which was one of the most interesting features in this case.

As mentioned before, most of the cases of KS arises in immunocompromised patients have an aggressive and fulminant course, whereas the relatively fair course and good response to treatment in our case might be in favor of the fact that the appearance of KS was not secondary to immunosuppression. It is worthy to note that occurrence of the second and third KS lesions in our case were 1 month after tapering off the prednisolone.

Altered immunity due to receiving immunosuppressive agents is not the only explanation for association of PV and KS. Although not proven yet, HHV‐8 has been introduced as a triggering factor of bullous disease, especially PV^23^, ^24^, ^25^ and there are some reports of detection of HHV‐8 virus on tissue samples obtained from pemphigus patients,^11^, ^25^, ^26^ even though data are contradictory in this regard.

Results of the recent cohort study showed that half of pemphigus‐associated KS cases developed their KS lesions before the start of immunosuppressive therapy that is fairly in favor of the role of HHV‐8 infection as a triggering factor in patients with pemphigus.^22^ However, these findings can be explained by considering KS as a result of Koebner phenomenon in patients with latent HHV‐8 infection.^26^ According to the above‐mentioned study, patients who developed KS lesions before initiation of immunosuppressive therapy showed a classic and limited form of disease, probably because of an activation of a latent HHV‐8 infection in patients with genetic susceptibility.^8^, ^22^

HHV‐8 detection in tissue samples of our patient could have shed some light on this matter of debate and suggested the pivotal role of HHV‐8 at least, in PV‐associated cases with a relatively fair course of disease, but regretfully, we could not test it which is a major limitation of this study.

The fact that tuberculin test in our patient was positive without any clinical manifestation of tuberculosis, might be inferred as the relative strength of cell‐mediated immunity that resulted in a benign course of KS in our case.

Treatment of KS depends on the clinical presentation, extent of involvement and associated comorbidities. In local diseases, radiation, photodynamic therapy, intralesional vinblastine, topical alitretinoin gel, cryotherapy, curettage and electrodesiccation are main therapeutic options. For extensive disease, a combination of surgery, chemotherapy and radiation is advocated. Some recent therapeutic agents such as imatinib and matrix metalloproteinase inhibitors are also being tried^27^, ^28^, ^29^ and some antiviral agents including ganciclovir, foscarnet, valganciclovir and cidofovir have been shown to have some therapeutic effects via inhibiting HHV‐8.^30^, ^31^ However, the best therapeutic approach for KS lesions in setting of PV is still remains to be elucidated. Although withdrawal of immunosuppressant agents seems to be the mainstay of management of these patients and could led to tumor regression in some cases,^32^ this may not always be feasible due to intractable nature of pemphigus in some patients. Hence, maintaining a suitable level of immunosuppression to sustain a balance between therapeutic and adverse effects of treatment in order to having both of diseases under controlled, is sometimes challenging and hard to achieve. As mentioned before, the occurrence of the second and third KS lesions in our case was 1 month after tapering off the prednisolone. This would challenge the theory considers the withdrawal of immunosuppressant agents as the best therapeutic modality in patients with pemphigus.^32^

In conclusion, we reported a case of KS with an unusual presentation to emphasize the fact that careful examination and clinical suspicion are the key factors for correct diagnosis. In fact, throat burning and odynophagia in PV patients might not be merely due to pemphigus erosions and should raise the suspicion of occurrence of other pathologic processes such as KS even in HIV‐negative patients.

Furthermore, we highlighted the fact that not all of patients with pemphigus‐associated KS have fulminant course. Therefore, the crucial question to be resolved is how we can discriminate between steroid‐associated KS and disease‐associated KS in patients with pemphigus.

Implementing some proceedings such as skin tuberculin test might be useful in answering this question and predicting the course of disease. Further studies are needed to shed more light on this topic.

## AUTHOR CONTRIBUTIONS

**Kamran Balighi:** Conceptualization (equal); supervision (equal); visualization (equal). **Maryam Ghiasi:** Data curation (equal); methodology (equal); project administration (equal). **Zeinab Aryanian:** Investigation (equal); writing – original draft (equal). **Zahra Nikyar:** Investigation (equal); project administration (equal); writing – original draft (equal). **Parvaneh Hatami:** Investigation (equal); visualization (equal); writing – review and editing (equal).

## CONFLICT OF INTEREST STATEMENT

The authors declare that they have no conflict of interest regarding the publication of this article.

## ETHICS STATEMENT

The patient provided written informed contest to publication of this case report and accompanying images.

## Data Availability

Data available on request due to privacy/ethical restrictions.
